# No time to rest: seasonal dynamics of non-structural carbohydrates in twigs of three Mediterranean tree species suggest year-round activity

**DOI:** 10.1038/s41598-021-83935-1

**Published:** 2021-03-04

**Authors:** Anna M. Davidson, Sylvia T. Le, Katelyn B. Cooper, Eden Lange, M. A. Zwieniecki

**Affiliations:** 1 Department of Plant Sciences, University of California Davis, Davis, CA 95616 USA; 2 Present Address: Department of Natural Resources and the Environment, Cornell University, Ithaca, NY 14853 USA

**Keywords:** Plant ecology, Plant physiology

## Abstract

Perennial plants in temperate climates evolved short and long-term strategies to store and manage reserves in the form of non-structural carbohydrates (NSC; soluble sugars (SC) and starch (St)). NSC storage allows plants to survive seasonal periods of photosynthetic inactivity (dormancy). To study year-to-year seasonal patterns of trees’ NSC dynamics that control phenology and yields, we established a large scale, multi-year study called the “Carbohydrate Observatory” using a citizen science approach with ~ 590 sites throughout the Central Valley of California. Monthly sampling tracked seasonal trends of starch and sugar levels in both xylem and phloem of twigs in *Prunus dulcis*, *Pistacia vera* and *Juglans regia.* Presented is the initial technical analysis of the first 3 years. With no exception, levels of reserves changed continuously throughout the year suggesting that even during dormancy, the average concentration of NSC, starch and sugars varies seasonally. In general, carbohydrate reserves are highest entering dormancy. During winter, NSCs slowly decrease to depletion during bloom time and remain low during summer until recovery near harvest. Starch is the major reserve compound in the wood of *P. dulcis* and *P. vera* while soluble sugars are the major reserves in *J. regia*. NSC content fluctuates throughout a season and significantly varies between years suggesting intrinsic and climatic effects on trees’ energy reserves.

## Introduction

Most basic plant functions, like growth, energy metabolism, maintenance respiration, osmosis and transport, are dependent on non-structural carbohydrates (NSC). Generally, NSC exist as two pools: chemically active soluble carbohydrates (SC) present as mono and disaccharide sugars like glucose, fructose, and sucrose and sometimes sugar alcohols like sorbitol, and inactive, insoluble starch (St)^1–3^. These two pools of carbohydrates are under biological control that evolved to meet demands of species-specific internal (physiology) and external (environmental) properties^4^. Allocation of NSC to the aforementioned functions must be balanced with storage in order to buffer asynchronous supply and demand on different temporal and spatial scales^5^. All plants evolved the capacity to overcome a daily cycle of discontinuous photosynthate availability by accumulating carbohydrate reserves to survive nights^6–13^. It is also known that perennial plants had to evolve long term strategies to acquire energy reserves as they cycle through periods of growth and reproduction (activity) that are associated with photosynthetic activity and periods of inactivity (dormancy)^4,14^.

These long-term strategies require that during the times of activity, NSC reserves are accumulated and allocated to processes that require energy or structural input such as growth, reproduction, and defense. To survive the continuous metabolic requirements during dormancy and later allow for the resumption of post dormancy growth (spring flowering and leafing), deciduous perennial plants must acquire and store an ample supply of carbohydrates in their woody tissue^1,15,16^. During bud break, fast and efficient mobilization of stored carbohydrates are needed to sustain bud growth^3,17,18^. Thus, the amount of NSC reserves ahead of the winter should be enough to sustain winter respiration and spring demands for bloom and leafing. Species vary in their seasonal pattern of NSC reserve pools based upon vegetative habit, reproduction, growth, and duration of dormancy^4,14^. These evolved NSC management traits are being challenged now by fast climatic changes such as the rise of global temperatures, the increase in the frequency of droughts, and the length and severity of winter with significant, often detrimental, effects on tree phenology^19–21^ endangering the future of orchards and natural ecosystems. Therefore, it is important to study inter-seasonality of NSC patterns, that occur during adverse conditions like low temperature or prolonged drought^10,22,23^.

Interestingly, during the active growing period, the pools of NSC are relatively low and constant while the highest variability of the NSC pools are observed during the time of dormancy and spring bloom^24^. As orchard trees were presumably selected for high productivity in expense of energy reserves security, even small changes to NSC management induced by climate change or management practices can disproportionally affect productivity and orchard health.

**Figure 1:**
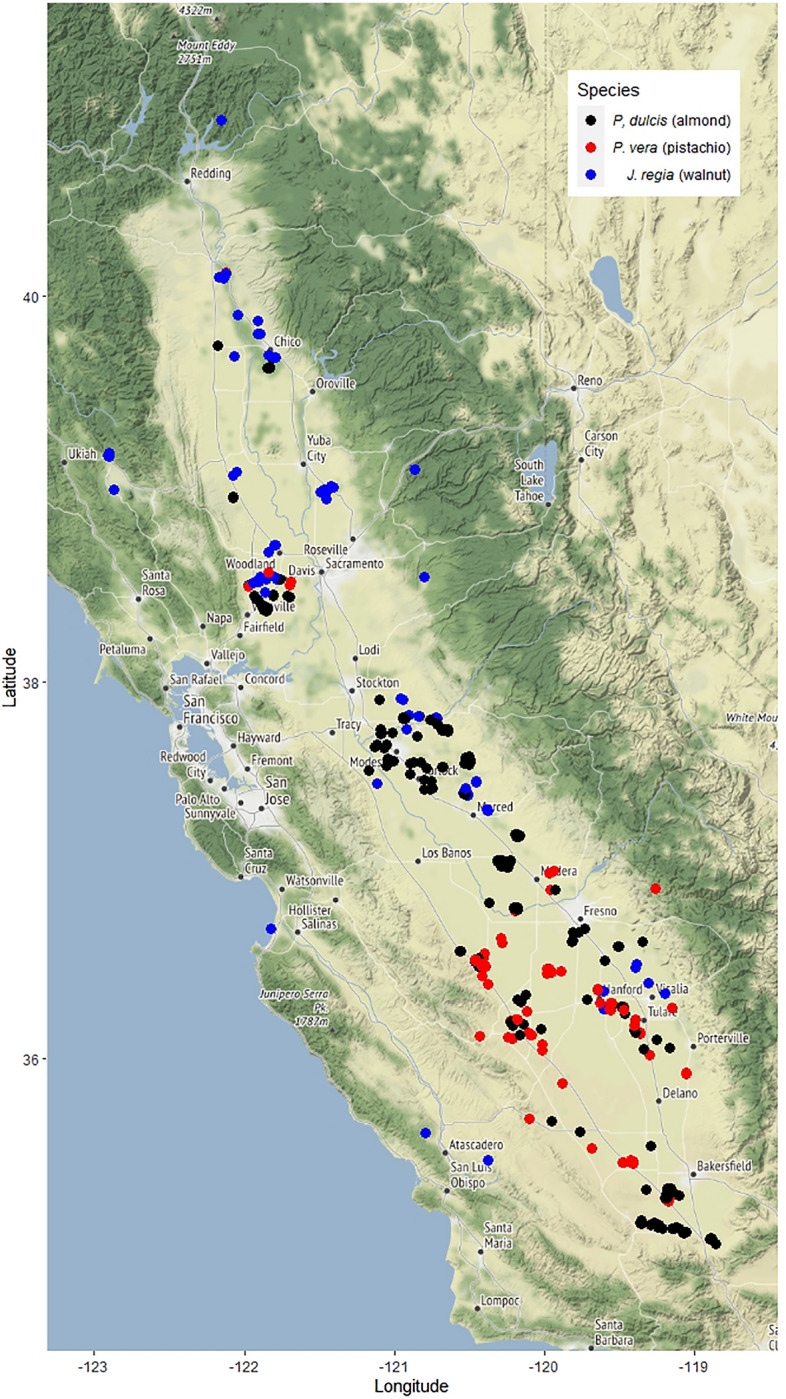
Map of the Central Valley of California depicting the locations of participating orchards of three species (*P. dulcis, J. regia, and P. vera*). Each point may represent multiple sites that are close in latitude and longitude (R version 3.6.3 package “ggplot2”^25^).

The conventional trend is that NSC reserves increase over the late growing season and decrease over the dormant season. Furze et al.^14^ conducted a detailed within-year NSC study at the organ and whole tree level of five dominant temperate tree species in the Northeast to quantify total NSC storage over the course of a year and found that in general, whole-tree total NSC pools built up over the growing season and declined over the dormant season. However, there are no long-term observational studies over multiple years that look at interannual variability. Thus, the questions of how active and variable the pool of carbohydrate reserves in trees are and when reserves are being used and replenished in respect to trees’ phenology and climatic conditions still remains open. Therefore, to begin to understand intra and inter-seasonal dynamics and patterns of tree NSC, a large scale, multi-year, multi-species study called the “Carbohydrate Observatory” was established in 2016, Using a citizen science approach, samples were obtained from ~ 590 orchard sites throughout the Central Valley of California (Fig. 1). This large-scale approach and monthly sampling allowed for the tracking of seasonal trends of starch and sugar levels in both xylem and bark from twigs of three major tree nut species: *Prunus dulcis, Pistacia vera* and *Juglans regia.* *An interactive map and* available NSC data are accessible via a dedicated website https://zlab-carb-observatory.herokuapp.com/. The objective of this paper is to present a general overview of a multi-year temporal pattern analysis of sugar, starch and total NSC dynamics between xylem and phloem tissues in three tree species in respect to their differing phenology and bloom habit over the 3-year period from January 2017 to December,-2019. *P. dulcis* is an insect pollinated perfect flower species, *J. regia* is a monoecious, wind pollinated species and *P. vera* is a dioecious wind pollinated species (Table 1). These seasonal trends of multiple species lays the framework for future phenology, climate, and management studies.

**Table 1:** Botany, phenology and origin of *J. regia*, *P. dulcis*, and *P. vera.*

Species	*J. regia*	*P. dulcis*	*P. vera*
Flowers	Monoecious, male catkins, female terminal flowers in groups of 2–5	Perfect on spurs	Dioecious
Height	25–30 m	4–10 m	10 m
Bloom/leafing	April	February	March
Harvest	October	August	August
Origin	Mountains of Asia	Iran, Turkey, Central Asia	Middle East, Central Asia

## Materials and methods

Using a Citizen Science approach, growers spanning the entire Central Valley of California sent samples of current-season wood and bark from twigs of *P. dulcis* (Mill. D.A Webb), *P. vera* L. and *J. regia* L. trees. This analysis spans a time period from January, 2017 to December 2019. We analysed 590 orchard sites, with a total of 5330 individual observations, split between *P. dulcis* (2502) *P. vera* (1781) and *J. regia* (1047). We encouraged growers to collect samples once a month, although frequency and participation level varied over time.

A specific unified protocol for sample collection required that one current season twig from three trees per orchard were cut at the base of the twig where the current season’s wood met last year’s wood. The bark from the lower 10 cm of the twig was removed using a razor blade. Both the bark and the wood of the three twigs were put in a paper envelope and mailed to the laboratory for NSC analysis. In total, we performed 31,980 soluble sugars and 31,980 starch analyses for bark and wood. It is important to note that prior to the project we tested the integrity of the NSC content over shipping time and found that sugar and starch do not break down within 4 days (the time longer then required for local mail to reach the laboratory from anywhere in California). Analysis of the NSC integrity was tested for each species on five separate twigs. Each twig was collected using the standard protocol and separated to four sections. One piece was immediately place in the oven while the remaining samples were left in the shipping envelopes. On day 2 another piece was removed from the envelope, similarly another sample was taken on day 3 and 4 (4 days is the maximum time samples are spent in shipping from anywhere in California before being placed in the drying oven). We used a linear mixed model with twig as random factor to determine presence of changes in NSC, SC and St content in wood and bark. There was no missing data. Results of the analysis are presented in Table 2. In no case, did the time spent in the envelope significantly affect the centration of NSC.

**Table 2:** Results of a linear mixed model analysis for the possible impact of time samples spent in a shipping envelope prior to drying.

Species	NSC form	Test results	Chisq	Df	Pr (> Chi sq.)
*J. regia*	SC	Issue	273.6452	1	< 2e − 16***
		Days	0.1810	1	0.6705
		Tissue:days	0.0075	1	0.9312
	Starch	Tissue	157.7377	1	< 2e − 16***
		Days	0.5476	1	0.4593
		Tissue:days	0.0061	1	0.9379
*P. dulcis*	SC	Tissue	157.7377	1	< 2e − 16***
		Days	0.5476	1	0.4593
		Tissue:days	0.0061	1	0.9379
	Starch	Tissue	26.7213	1	2.35e − 07***
		Days	2.1121	1	0.1461
		Tissue:days	0.8540	1	0.3554
*P. vera*	SC	Tissue	2.8176	1	0.0033935**
		Days	1.1492	1	0.484893
		Tissue:days	7.1942	1	0.1302
	Starch	Tissue	348.7246	1	< 2e − 16***
		Days	0.5559	1	0.4559
		Tissue:days	0.5625	1	0.4532

No significant impact up to 4 days was found between immediate and delayed drying in concertation of starch and soluble sugars for any of the three species.

Significance codes: 0 ‘***’ 0.001 ‘**’ 0.01 ‘*’.

Upon arrival, samples were put in the dryer for 48 h at 75 °C. The bark and wood were chopped into small < 1 mm pieces separately and ~ 100 mg of each were ground into a fine powder (~ 1 µm) using a ball grinder (MiniBeadbeater-96, Glen Mills Inc., NJ). To analyse for sugar and starch we used the previously described protocol from Leyva et al*.*^26^; with modifications described in Tixier et al.^24^. Specifically, 25 mg of powder per sample was placed in 1.5 ml tubes. Tubes were then treated with 1 ml of sodium acetate buffer (0.2 M, pH 5.5), vortexed, and incubated in a 70 °C water bath for 15 min and centrifuged (10 min at 21,000*g*). 50 µl of supernatant was extracted and diluted in ultra-pure (UP) water (1:20 v:v) and vortexed. SC content was quantified from diluted supernatant tubes using an anthrone/sulfuric acid colorizing reagent (0.1% (m:v) in 98% sulfuric acid) and reading absorbance at 620 nm in a spectrophotometer. The remaining centrifuged tubes with the pellet and buffer were used for starch quantification. To extract starch, the tubes were vortexed to suspend the pellet, boiled at 100 °C for 10 min to allow starch gelatinization and let sit for 20 min at room temperature (22 °C). Once cooled, 100 µl of amyglucosidase (7 units/ml^−1^, Sigma-Aldrich) and 100 µl amylase (0.7 units/ml^−1^, Sigma-Aldrich) were added to the tubes and incubated for 4 h at 37 °C in a rotating incubator. Samples were then centrifuged (10 min at 21,000*g*). 50 µl of supernatant was extracted and diluted in 1 ml UP water (1:20 v:v) and vortexed. Total soluble sugar content was analysed using the same method described above. Starch content was determined by subtracting original SC content from post-digestion SC content. All samples were plated on 96-well plates. To account for any procedural variability (chemicals, timing, pipetting, temperature etc.), each plate contained a glucose standard curve and wood/bark standard tissue samples with known SC and St content (four per plate) that were concurrently undergoing all steps in the same chemical analysis. The wood/bark standard tissue is a sample from a homogenous mix of several thousand ground samples leftover from a 2016 preliminary part of the study. We measured out 25 mg of 96 samples at of the mix and analyzed them for NSC (starch and sugar) using our protocol.

For statistical analysis we used R. For Kruskal Wallis using ‘kw’ function from R package ‘seastests v0.14.2’ with 12 months lag time. For the augmented Dickey Fuller (ADF) test we used ‘adf.test’ function from R package ‘tseries v0.10-47’. For ANOVA analysis to compare carbohydrate content between months in consecutive years, we used the ‘aov’ function from R package ‘stats v3.6.2’.

## Results

### Analysis of seasonality in carbohydrate content

In all three species, there was a general pattern of seasonal changes in carbohydrate content. The Kruskall Wallis test for seasonality (presence of seasonal periodicity) suggests that, with the only exception of soluble sugar content in the wood of *J. regia*, all NSC, St, and SC in bark and wood in all three species showed significant seasonal trends with a 12-month frequency (annual cycle) (Table 3). To determine the possibility of the unit root presence (test for stationarity, i.e. presence of significant interannual variation) in a time series, we used the Augmented Dickey Fuller (ADF) test. Based on the analysis we cannot reject the null hypothesis that there is a unit root for all three species, and NSC location/types. In all cases, the ADF test resulted in p > 0.05% (Table 4), thus suggesting that our time series may not be stationary and that there is a year-to-year variation. This technical analysis suggests the presence of unaccounted for, stochastic year-to-year variation and indicate need for future study to determine the origin of the variation. To determine which months were most affected by climatic variability, we used analysis of variance to compare carbohydrate content for particular months between years (Table 5). Generally, this analysis suggests the presence of significant differences in *P. dulcis* and *P. vera* for both SC and St and to some extent in *J. regia* in the levels of sugars but not starch (Table 5).

**Table 3:** Kruskall Wallis test for seasonality in a time series with lag time of 12 months.

	Total NSC	Wood NSC	Bark NSC	Wood SC	Wood starch	Bark SC	Bark starch
*P. dulcis*	*0.0007207*	*0.0007065*	*0.0120546*	*0.0035869*	*0.0007535*	*0.0074689*	*0.0026537*
*P. vera*	*0.0011088*	*0.0013211*	*0.0017527*	*0.013528*	*0.0008455*	*0.0160282*	*0.0007386*
*J. regia*	*0.0097848*	*0.0225551*	*0.0046629*	0.1858203	*0.0035793*	*0.0282376*	*0.0050683*

Italics denote p < 0.05—significant seasonality.

**Table 4:** Augmented Dickey Fuller (ADF) test for unit root in time series with lag time of 12 months.

	Total NSC	Wood NSC	Bark NSC	Wood SC	Wood starch	Bark SC	Bark STARCH
*P. dulcis*	0.576	0.587	0.962	0.779	0.6517	0.975	0.701
*P. vera*	0.645	0.627	0.929	0.980	0.573	0.989	0.971
*J. regia*	0.887	0.729	0.941	0.727	0.920	0.762	0.474

**Table 5:** Results of ANOVA analysis for presence of significant difference in content of carbohydrates between months in consecutive years.

Month	Total NSC	Wood NSC	Bark NSC	Wood SC	Wood starch	Bark SC	Bark starch
***P.dulcis***							
Jan	*7.083E − 05*	*1.17E − 02*	*1.77E − 11*	*1.63E − 08*	*4.94E − 02*	*4.78E − 16*	7.11E − 01
Feb	0.0769662	5.58E − 01	*4.00E − 06*	*2.91E − 04*	6.27E − 01	*3.69E − 09*	1.75E − 01
Mar	*0.0007313*	*2.44E − 02*	*2.58E − 07*	*1.74E − 09*	9.56E − 01	*8.15E − 11*	9.64E − 01
Apr	*0.0005371*	*4.29E − 02*	*9.29E − 10*	*1.12E − 11*	9.13E − 01	*4.21E − 11*	6.98E − 01
May	*0.0492458*	6.38E − 01	*2.98E − 06*	*4.94E − 06*	2.00E − 01	*9.04E − 09*	9.26E − 01
Jun	0.1439405	7.55E − 01	*1.46E − 07*	*5.85E − 06*	1.29E − 01	*5.32E − 10*	4.40E − 01
Jul	0.1606736	8.49E − 01	*5.23E − 06*	*2.02E − 07*	5.00E − 02	*5.36E − 10*	8.51E − 01
Aug	*0.0164891*	4.66E − 01	*2.10E − 09*	*1.82E − 04*	8.72E − 01	*6.35E − 09*	6.73E − 02
Sep	0.0574218	5.36E − 01	*1.36E − 05*	*5.39E − 06*	4.95E − 01	*3.71E − 09*	4.01E − 01
Oct	*0.0010045*	*1.64E − 02*	*1.25E − 08*	*5.80E − 05*	1.21E − 01	*1.70E − 10*	7.12E − 01
Nov	*0.0180753*	5.64E − 02	*2.45E − 04*	*4.17E − 04*	3.84E − 01	*3.23E − 06*	8.08E − 01
Dec	*0.0095215*	1.82E − 01	*3.31E − 07*	*2.24E − 03*	7.24E − 01	*4.65E − 08*	3.55E − 01
***P. Vera***							
Jan	7.12E − 02	1.35E − 01	*6.30E − 03*	1.16E − 01	2.08E − 01	*4.70E − 03*	5.02E − 02
Feb	2.57E − 01	2.85E − 01	2.76E − 01	*1.25E − 02*	3.26E − 01	3.63E − 01	5.14E − 01
Mar	*4.66E − 02*	7.18E − 02	*8.99E − 03*	*1.16E − 04*	5.05E − 01	*4.00E − 04*	7.52E − 01
Apr	3.19E − 01	5.83E − 01	*2.18E − 02*	*9.24E − 03*	9.46E − 01	1.15E − 01	1.33E − 01
May	*4.95E − 02*	9.53E − 02	*1.73E − 02*	*9.43E − 04*	4.70E − 01	*5.97E − 03*	5.44E − 01
Jun	9.38E − 02	2.59E − 01	*1.30E − 03*	*2.94E − 02*	5.04E − 01	*1.20E − 02*	6.38E − 02
Jul	6.30E − 02	1.57E − 01	*1.45E − 03*	*1.65E − 03*	6.20E − 01	*6.00E − 05*	1.88E − 01
Aug	*2.05E − 02*	*3.80E − 02*	*1.24E − 02*	*2.03E − 04*	2.65E − 01	*1.62E − 03*	6.44E − 01
Sep	*1.48E − 02*	*3.30E − 02*	*4.26E − 03*	*3.34E − 03*	1.14E − 01	*5.58E − 03*	*3.67E − 02*
Oct	6.51E − 01	7.87E − 01	*3.97E − 02*	3.65E − 01	9.64E − 01	*2.63E − 03*	7.12E − 01
Nov	1.65E − 01	2.89E − 01	1.33E − 01	*1.32E − 02*	8.53E − 01	2.95E − 01	6.02E − 01
Dec	*1.54E − 02*	*3.23E − 02*	*4.23E − 03*	*1.12E − 03*	1.85E − 01	*3.33E − 03*	*4.97E − 02*
***J. regia***							
Jan	5.17E − 01	2.09E − 01	9.91E − 01	9.12E − 01	1.93E − 02	*3.31E − 02*	*2.53E − 02*
Feb	3.46E − 01	6.88E − 01	*4.94E − 02*	3.83E − 01	7.55E − 01	*1.92E − 02*	6.90E − 01
Mar	5.00E − 02	*1.38E − 02*	1.38E − 01	3.90E − 01	*1.50E − 03*	8.49E − 01	*1.04E − 02*
Apr	2.88E − 01	2.89E − 01	8.88E − 02	3.85E − 01	1.39E − 01	*1.27E − 04*	5.79E − 01
May	2.98E − 01	5.07E − 01	1.55E − 01	1.20E − 01	7.01E − 01	*3.04E − 04*	2.01E − 01
Jun	4.94E − 01	4.55E − 01	4.75E − 01	2.94E − 01	4.64E − 01	*3.44E − 02*	4.50E − 01
Jul	2.05E − 01	1.41E − 01	4.56E − 01	3.53E − 01	*2.80E − 04*	1.43E − 01	*9.43E − 03*
Aug	1.31E − 01	*1.91E − 02*	6.81E − 01	5.38E − 01	*8.47E − 04*	*2.68E − 03*	*3.96E − 02*
Sep	3.62E − 01	1.68E − 01	1.59E − 01	3.23E − 01	1.30E − 01	*1.60E − 02*	4.57E − 01
Oct	6.94E − 01	3.69E − 01	8.40E − 01	7.29E − 01	2.73E − 01	3.03E − 01	5.52E − 01
Nov	9.21E − 01	7.42E − 01	9.32E − 01	9.37E − 01	1.81E − 01	2.13E − 01	4.03E − 01
Dec	5.80E − 01	5.83E − 01	5.13E − 01	1.50E − 01	8.54E − 02	7.29E + 00	8.54E − 01

Italics denotes p < 0.05.

### Total NSC

**Figure 2:**
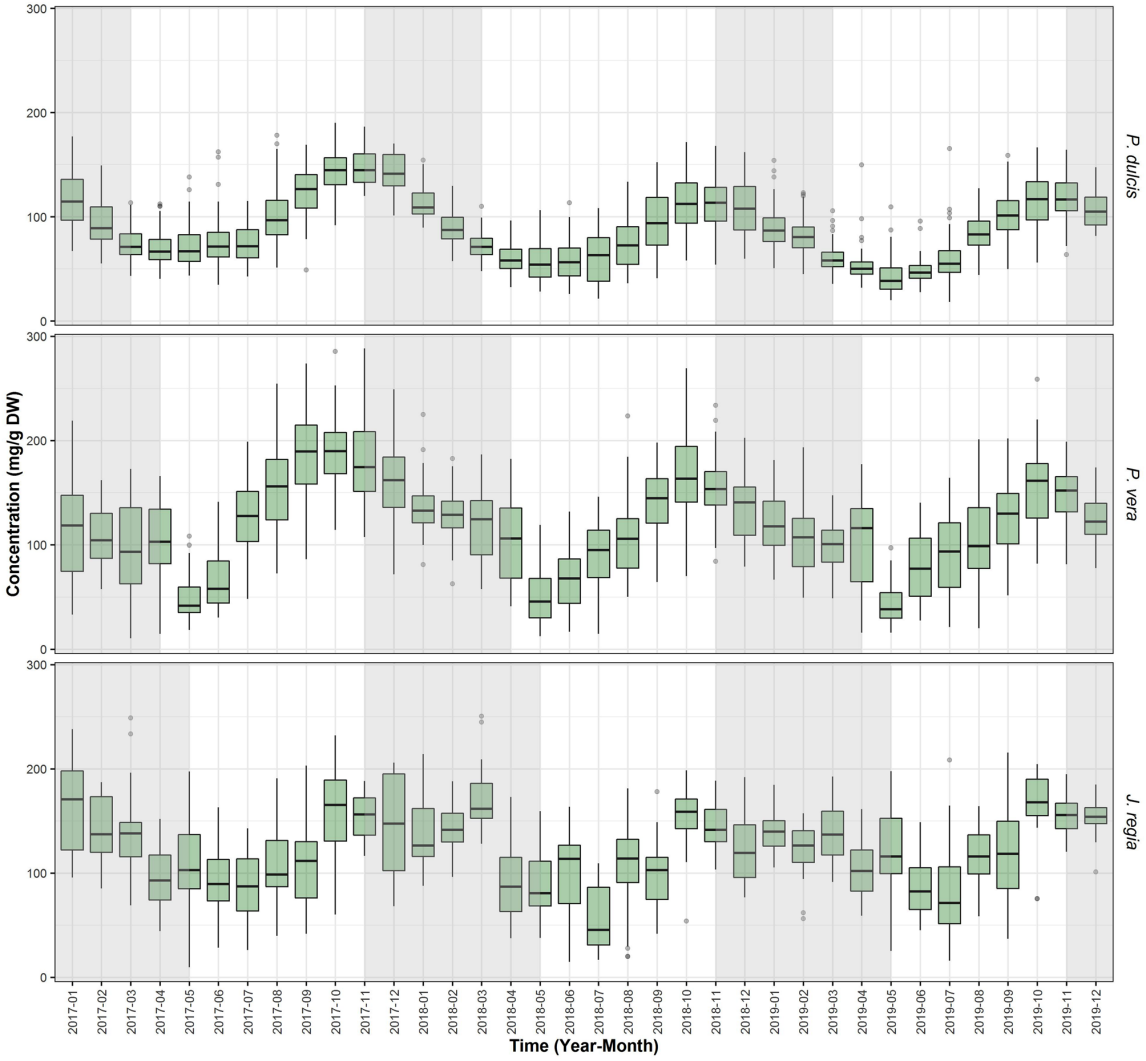
Multiyear seasonal trends of total NSC of three species. Average concentration of total NSC (SC + St) from all sites in California for *P. dulcis, P. vera*, and *J. regia* from January 2017 to December 2019. The lower and upper hinges correspond to the first and third quartiles (the 25th and 75th percentiles). The upper whisker extends from the hinge to the largest value no further than 1.5*IQR from the hinge (where IQR is the inter-quartile range, or distance between the first and third quartiles). The lower whisker extends from the hinge to the smallest value at most 1.5*IQR of the hinge. Grey shading reflects the dormancy period of each species.

In all three species, the average NSC content varies seasonally with species-specific patterns (Fig. 2). In general, NSC content expressed in mg of NSC per 1 g of twig dry mass (mg/g DW) was highest in *P. vera* and lowest in *P. dulcis*. The average maximum NSC content was observed in fall months (September to October) preceding the typical leaf senescence and ranged from 113 to 148 mg/g DW in *P. dulcis*, 156–189 mg/g DW for *P. vera,* and 154–165 mg/g DW in *J. regia*. These high levels were followed by the dormancy period during which, NSC content in branches is depleted prior to spring bud break. Throughout the bud break period, NSC depletion was continuous in *P. dulcis* but in both *P. vera* and *J. regia* we observed a characteristic increase in NSC content in April/May that coincides with leafing. Lowest levels of NSC were observed in April and May in *P. dulcis* (45–71 mg/g DW), May in *P. vera* (45–52 mg/g DW) and July in *J. regia* (58–84 mg/g DW), which typically coincides with the most active period of vegetative growth (diameter and height) and initial nut filling. From June to July we observed a recovery of the NSC content to maximum in the fall (Fig. 2). This pattern was repeated every year, although levels of carbohydrate reserves for *P. dulcis* and *P. vera* were significantly different in consecutive years (Table 3) especially during winter. There were no significant differences in total NSC content in *J. regia*.

### NSC in wood and bark

**Figure 3:**
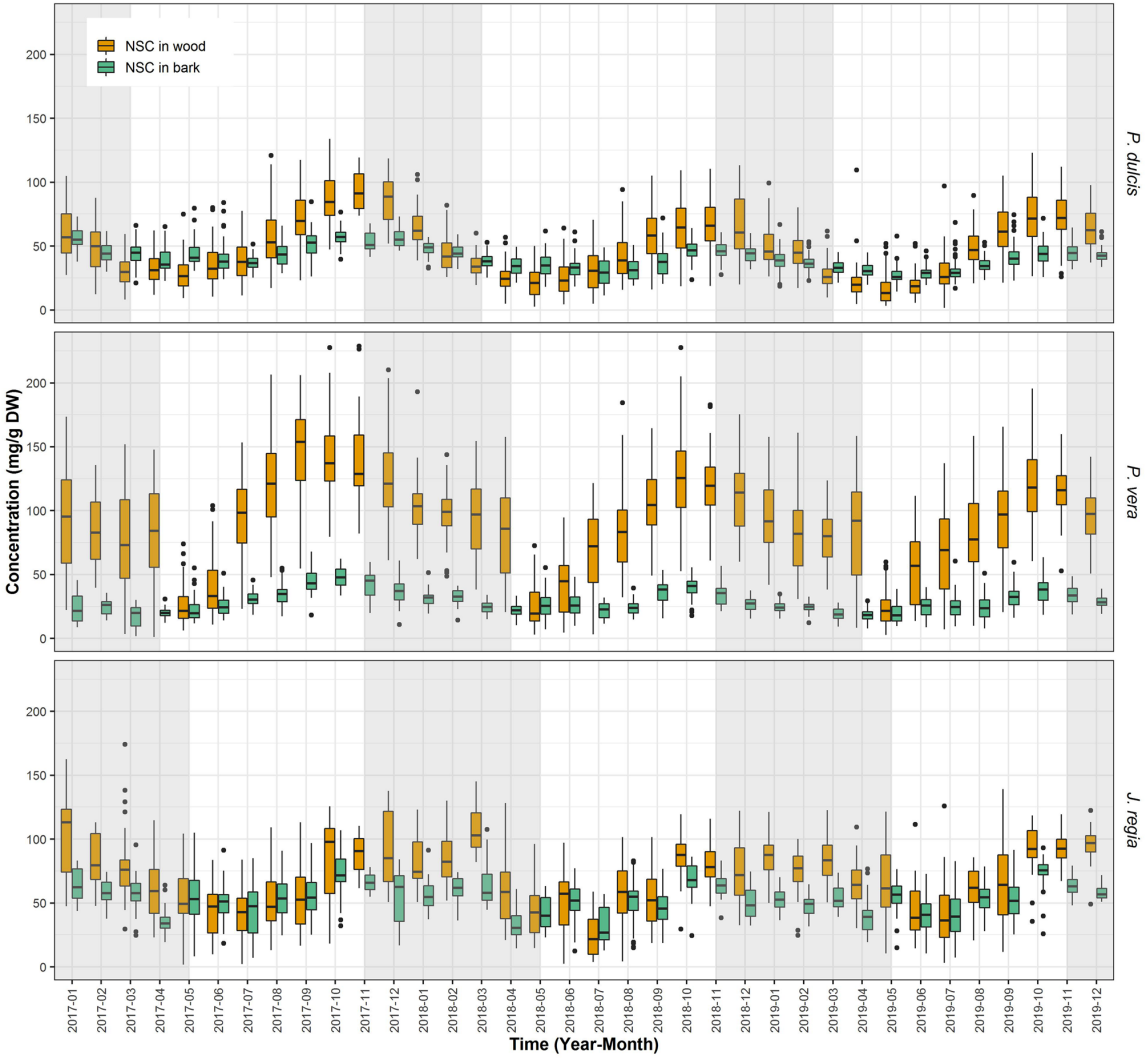
Total NSC concentration in wood and bark in three species. Average concentration of total NSC (Sc + ST) in wood (orange) and in bark (green) from all sites for *P. dulcis*, *P. vera* and *J. regia* from January 2017 to December 2019. The upper whisker extends from the hinge to the largest value no further than 1.5*IQR from the hinge (where IQR is the inter-quartile range, or distance between the first and third quartiles). The lower whisker extends from the hinge to the smallest value at most 1.5*IQR of the hinge. Grey shading reflects the dormancy period of each species.

In twigs, bark (cork, phloem and cambium), and xylem are tissues that have the capacity to store NSC. For all three species, the general pattern of NSC content is similar to each other with maximum in the fall and minimum in the early summer (Fig. 3). NSC content was expressed in mg per gram of twig dry weight and adjusted for the contribution of bark and wood to total twig dry mass such that the sum of the NSC in bark and wood equals the total NSC content provided on Fig. 2. In all three species, seasonal changes of NSC content is much higher in the wood than in bark, with content in the wood being lower or equal to that observed in bark during summer and higher to that observed in the fall. This pattern was most pronounced in *P. vera* where NSC content in wood was three times higher than in bark during October (~ 120 to ~ 40 mg/g DW). In *P. dulcis* wood NSC content was 1.5 times higher (~ 77 to 49 mg/g DW) in the fall than in bark and in *J. regia* NSC content in the wood was only 1.3 times higher than in bark (~ 89 to 68 mg/g DW). Those differences were very consistent in consecutive years. Absolute content of NSC in wood was relatively constant for each month between years in all three species. However, content in the bark varied significantly among the years in *P. vera* and *P. dulcis* but was invariable in *J. regia* (Table 3).

### Starch and soluble sugars in bark

**Figure 4:**
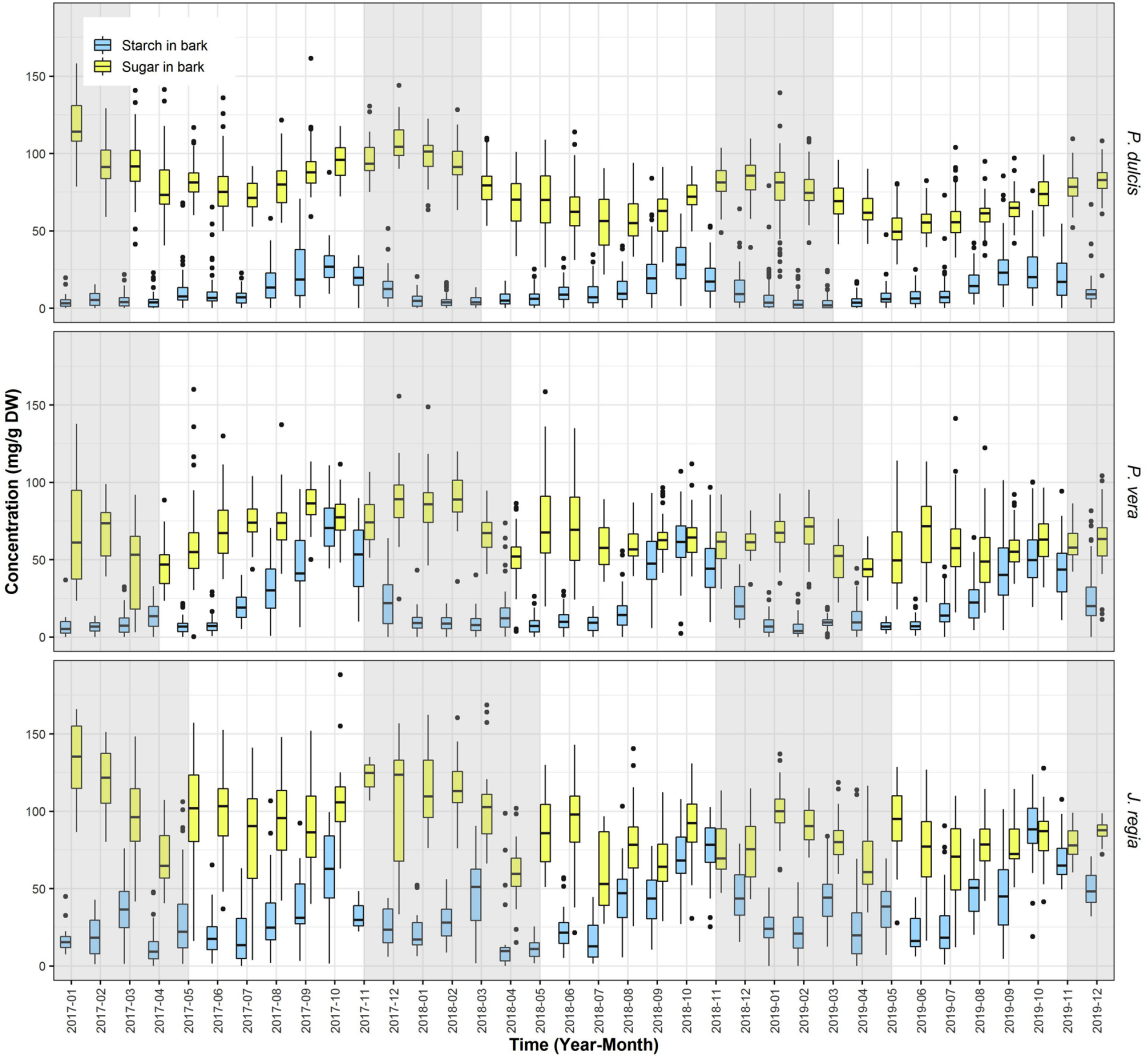
Starch and sugar in bark. Average concentration of starch (blue) and sugar (yellow) in bark from all sites for *P. dulcis*, *P. vera* and *J. regia* from January 2017 to December 2019. The upper whisker extends from the hinge to the largest value no further than 1.5*IQR from the hinge (where IQR is the inter-quartile range, or distance between the first and third quartiles). The lower whisker extends from the hinge to the smallest value at most 1.5*IQR of the hinge. Grey shading reflects the dormancy period of each species.

Soluble sugars were the dominant form of NSC reserves in the bark of all three species (Fig. 4). In *P. dulcis* SC content varied from 23 to 53 mg/g DW and St content varied from 1.7 to 13 mg/g DW, in *P. vera* SC content varied from 14 to 29 while St content varied from 2 to 23 mg/g DW, and in *J. regia* SC content varied from 26 to 57 with St varying from 4 to 37 mg/g DW. SC content was less variable than in the wood tissue with variation being only ~ 2 times different between low content in spring and high in winter. St content was much more variable. During summer, levels of St reserves in the bark were near complete exhaustion (near < 5 mg/g DW) but recovered by fall (September–October) contributing significantly to the NSC reserves. Surprisingly, the bark St reserves were depleted before the winter (by December) and remain low through the rest of the dormancy period. This pattern was highly consistent across the 3 years of observations and similar in all three species. Year-to-year SC content variation in bark was significant in all three species while starch content only varied in *J. regia* (Table 3).

### Starch and soluble sugars in wood

**Figure 5:**
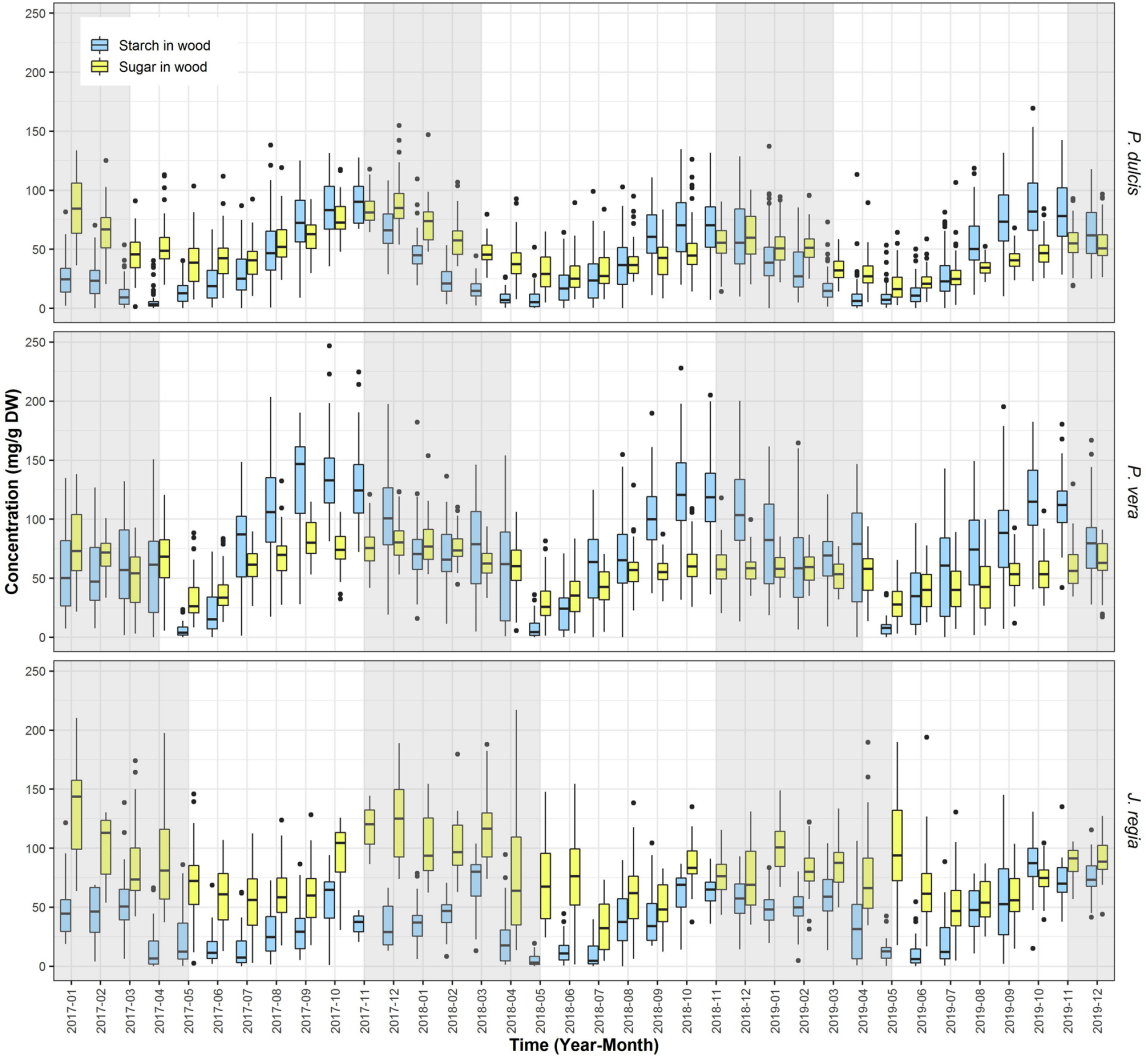
Starch and sugar in wood. Average concentration of starch (blue) and sugar (yellow) in wood from all sites for *P. dulcis*, *P. vera* and *J. regia* from January 2017 to December 2019. The upper whisker extends from the hinge to the largest value no further than 1.5*IQR from the hinge (where IQR is the inter-quartile range, or distance between the first and third quartiles). The lower whisker extends from the hinge to the smallest value at most 1.5*IQR of the hinge. Grey shading reflects the dormancy period of each species.

Both SC and St content in the wood undergo significant seasonal fluctuation. All three species seemed to follow similar patterns. Highest levels of soluble sugars and starch occurred in the fall and lasted through the winter, while lowest levels of starch and soluble sugars occurred during spring and early summer (Fig. 5). In *P. dulcis* SC content varied from 10 to 48 mg/g DW and St content varied from 3.5 to 48 mg/g DW, in *P. vera* SC content varied from 19 to 57 mg/g DW while St content varied from 4 to 92 mg/g DW, and in *J. regia* SC content varied from 19 to 76 mg/g DW with St varying from 3 to 50 mg/g DW. In general, starch provides a much higher contribution to NSC content in wood than in bark and in *P. vera* and *P. dulcis* wood starch is the dominant form of NSC reserves during winter. Year-to-year variation was highly significant in the wood only for soluble sugars in *P. vera* and *P. dulcis* and non-variable in *J. regia*. Starch content, on the other hand, remained very invariable between years in *P. dulcis* and *P. vera*, but was significantly different for several months in *J. regia* (Table 3).

## Discussion

Here we present unique multi-year pattern analyses of sugar and starch in wood and bark from three major nut species covering a large geographical scale spanning the Central Valley of California. We focused our analysis on NSC content in twigs. Compared to other parts of the tree, twig NSC content follows a general pattern of NSC seasonal variation^14,27^ and correlates with or even affects major phenological events^28^ whereas roots remain fairly stable throughout the year Additionally, despite the stem wood having the biggest biomass, branches are the largest reservoir of total NSC followed by roots and stem wood^14^. Sampling of twigs is also non-invasive for trees and allows for easy and fast collection of samples by the citizen scientists.

### Seasonality

The seasonal patterns of NSC, starch, and sugar show global similarities and some species-specific differences. One major result of this study is that NSC content in twigs is highly variable throughout the season with fall content being two-to-three times higher than during the active time of spring growth. The highest level of NSC reserves occurs between September and October, typically preceding leaf senescence by 1 or 2 months. This peak is followed by a continuous slow decrease of NSC content throughout the winter. These findings are consistent with other seasonal NSC studies. Matinez-Vilalta^4^ found that total NSC, starch, and soluble sugars varied seasonally, with a strong depletion of starch during the growing season and a general increase during winter months particularly in boreal and temperate biomes. Likewise, Furze et al.^14^ found pools built up over the growing season and declined over the dormant season. Specifically, they also found that NSC peaked in October for deciduous species. Variation in NSC content is continuous and significant changes in NSC content occurs during winter. This continuous reserve of carbohydrate content and form (SC or St) fluctuation suggests year-round activity, supporting the notion that there is no true dormancy. There is no time to rest for tree carbohydrate metabolism.

In all three species in all 3 years, accumulation of NSC reserves in twigs starts in mid-summer, during the nut filling period and active girth growth^27^ seemingly during the highest level of overall demand. This suggests that starch reserve accumulation is not a passive activity occurring in response to an excess of soluble sugars but is instead a competitive process to other carbon demanding processes like growth and fruit development^29^. Starch content reaches a maximum in bark in late summer (August–September) and in wood (September–October) and is followed by slow degradation while soluble sugars still accumulate in both tissues over winter. This suggests that starch degradation (as starch accumulation) is not a passive response to a low soluble sugar content level, but rather an active process performing biological functions potentially related to chill response and bloom timing^28^. The large swings of SC concentration in summer and winter and the counterintuitive response of St content might also suggest that SC levels are controlled at different concentrations in trees during the active period and dormancy. Interestingly, we did not observe any spike in NSC accumulation postharvest when one would expect an excess of photosynthates due to removal of fruits and significant slowdown in growth while maintaining leaf photosynthetic capacity. However, we only analyzed the twigs, thus it is possible that, at the end of the tree growth cycle during fall, most of the photosynthetic output is exported to basal parts of the tree–trunk and roots^27^.

During winter, a slow loss of total NSC observed in all three species is accompanied by highly dynamic changes in soluble sugar and starch content. The slow loss could be explained by respiration activity that is affected by winter temperatures^30^ and possibly whole tree redistribution of sugars^1,24^. However, the large swings between NSC forms needs a deeper look. Starch degradation over winter seemed to be associated with an increase of SC that lasts till bloom/leafing. The increase in SC during winter is often explained as part of the tree’s response to frost that requires input of osmotica and energy to counter frost, but in the Central Valley in California, frost is a highly infrequent event and cannot be used to explain the observed trend. It is more likely that accumulation of SC and degradation of starch reflects underlying biological activity where SC-starch balance during winter can be associated with time/temperature memory of dormancy^28^ and could be linked to the concept of chilling in future studies.

The three species differed in variation between minimum and maximum NSC content: *P. vera* has the most yearly variation of ~ 300%, *P. dulcis* ~ 200%, while *J. regia* only ~ 150%. It seems that this yearly variation is not related to maximum pre-winter reserves (as maximum NSC was similar for all three species), but rather minimum amounts tolerated by each species in the summer. Thus, pre-winter accumulation of NSC that could reflect climatic constraints related to dormancy length seemed of lesser importance in seasonal patterns of NSC. However, tolerance to summer risks and yield capacity can be more important in shaping seasonal variation of NSC content. *P. vera* and *P. dulcis* seemed to tolerate very low reserve levels (risk taking) in late spring but rebuilt the reserves in competition to fruit and vegetative growth enforcing the notion of reserves being a strong competitive sink during summer in preparation for dormancy. *J. regia* reached a minimum of NSC in mid-summer before restoration of reserves is initiated allowing only a short period of time for reserves to build up. Overall though, *J. regia* seemed to maintain higher NSC levels in summer, potentially representing a lower risk strategy to summer stress.

### Year to year variation

In general, NSC, SC, and St content in wood and bark show highly significant seasonality in addition to significant interannual variation that suggests that while a general NSC pattern is most likely driven by seasonal/phenological events^29^, the year-to-year variation might be related to some stochastic parameters, potentially weather variation, tree breeding patterns (masting) and/or horticultural practices. However, this study spans only spans 3 years. The determination of the non-stationarity (year-to-year variation) using climatic (rainfall, temperature, solar time, bee-time etc.), management (changes in applied management strategies) or tree species-specific intrinsic responses (alternate bearing, phenology etc.) is premature, but possible in the near future.

While the annual pattern of NSC content in twigs, their distribution between bark and wood, and the dynamics of a starch and soluble sugar fraction showed high levels of seasonality and a similar pattern in three consecutive years, the absolute content shows some variability between specific months in a year-to-year analysis (patterns were non-stationary). This was especially true for *P. dulcis* and *P. vera* which show significant variation between months in the year-to-year analysis. The variations were especially pronounced in SC content. Interestingly, starch content was less variable suggesting that low summer values are persistent in the current climate under applied management practices, while high winter values potentially reflect physical space limitation for starch storage in twigs. In addition, year-to-year levels of variation of SC might be a result of not only climatic variability, but also yield history especially in alternate bearing species. Nevertheless, assuming those levels of NSC and their composition is physiologically important; the year-to-year variation can provide useful information to assess orchard performance, potential yield, and orchard health.

The presented study provides a temporal analysis of NSC reserves in perennial cropping systems and opens a door for designing NSC-based novel management practices. Specifically, a better understanding of the post-harvest recovery of NSC content and its redistribution is vital as the tree prepares for dormancy. Perhaps the timing of irrigation, fertilization and other management practices should be carefully considered for this period in order to most efficiently recharge carbohydrate storage for the following season. Current practices like chemical defoliation might result in unexpected side effects related to their effect on fall NSC management. For example, reduced fall photosynthates supply may not affect NSC content in twigs but significantly reduce their export to trunk and roots affecting spring growth. Further, knowledge that *P. dulcis* and *P. vera* are running early summer growth and fruit set with almost zero reserves illustrates that this is the most vulnerable period, as even a small level of stress could result in carbon deficit and affect carbon demanding processes causing fruit drop, reduced vegetative growth or slowdown in NSC reserves buildup. The fact that there is no time to rest for carbohydrate management in trees underlines the importance of winter conditions on carbohydrate activity—their redistribution and twig preparation for bloom. Knowledge on minimum requirements to survive winter to assure synchronous bloom can offer new options to mitigate changing climatic conditions when evolved traits might be inadequate for new challenges like winter drought and extreme temperature swings. In addition, observed year-to-year variation in total NSC content and minimum/maximum values might have an impact on species productivity and explain yield variability. This unique large-scale multiyear study on both bark and wood dynamics and both intra and inter-seasonality variation, provides a solid foundation for future studies analyzing effects of climate and phenology.
